# Application of Raman Spectroscopy in Non-Invasive Analysis of the Gut Microbiota and Its Impact on Gastrointestinal Health

**DOI:** 10.3390/diagnostics15030292

**Published:** 2025-01-26

**Authors:** Patrycja Krynicka, George Koulaouzidis, Karolina Skonieczna-Żydecka, Wojciech Marlicz, Anastasios Koulaouzidis

**Affiliations:** 1 Department of Gastroenterology, Pomeranian Medical University, 71-252 Szczecin, Poland; krynickapatrycja@gmail.com (P.K.); wojciech.marlicz@sanprobi.pl (W.M.); 2 Department of Biochemical Sciences, Pomeranian Medical University, 71-460 Szczecin, Poland; koulaou@yahoo.co.uk (G.K.); karolina.skonieczna.zydecka@pum.edu.pl (K.S.-Ż.); 3 Department of Clinical Research, University of Southern Denmark, 57000 Odense, Denmark

**Keywords:** Raman spectroscopy, gut microbiota, gastrointestinal health, non-invasive diagnostics, microbial metabolites, short-chain fatty acids, personalised medicine

## Abstract

The gut microbiota, a complex community of microorganisms, plays a crucial role in gastrointestinal (GI) health, influencing digestion, metabolism, immune function, and the gut–brain axis. Dysbiosis, or an imbalance in microbiota composition, is associated with GI disorders, including irritable bowel syndrome (IBS), inflammatory bowel disease (IBD), and colorectal cancer (CRC). Conventional microbiota analysis methods, such as next-generation sequencing (NGS) and nuclear magnetic resonance (NMR), provide valuable insights but are often expensive, time-consuming, and destructive. Raman spectroscopy (RS) is a non-invasive, cost-effective, and highly sensitive alternative. This analytical technique relies on inelastic light scattering to generate molecular “fingerprints”, enabling real-time, marker-free analysis of microbiota composition and metabolic activity. This review explores the principles, sample preparation techniques, and advancements in RS, including surface-enhanced Raman spectroscopy (SERS), for microbiota research. RS facilitates identifying microbial species, analysing key metabolites like short-chain fatty acids (SCFA), and monitoring microbiota responses to dietary and therapeutic interventions. The comparative analysis highlights RS’s advantages over conventional techniques, such as the minimal sample preparation, real-time capabilities, and non-destructive nature. The integration of RS with machine learning enhances its diagnostic potential, enabling biomarker discovery and personalised treatment strategies for GI disorders. Challenges, including weak Raman signals and spectral complexity, are discussed alongside emerging solutions. As RS technology advances, mainly through portable spectrometers and AI integration, its clinical application in microbiota diagnostics and personalised medicine is poised to transform GI healthcare, bridging microbiota research with practical therapeutic strategies.

## 1. Introduction

The gut microbiota constitutes a complex bacterial, viral, fungal, and archaeal group crucial for maintaining gastrointestinal (GI) health and general well-being. Microorganisms contribute to significant host tasks like digestion, metabolism, and immune homeostasis. Furthermore, they provide neuroendocrine functions, which are all necessary for physical and mental balance. The gut microbiota initiates bioactive compounds, namely short-chain fatty acids (SCFAs, like acetate, propionate, and butyrate), by fermenting dietary elements that were not digested. As mentioned, SCFAs supply energy to colon cells, increase gut barrier integrity, and adjust inflammation. Moreover, microbiota synthesises crucial vitamins, including K and B, that further help general health [1].

The microbiota should be understood as a significant integrity modulator of the gut barrier through its association with increased mucus production and the structural and functional tightening of cell connections [2]. Variations in the microbiota composition and function, frequently named dysbiosis, are related to GI disorders, such as irritable bowel syndrome (IBS), inflammatory bowel disease (IBD), functional dyspepsia (FD), colorectal cancer (CRC), and systemic diseases like obesity and type 2 diabetes mellitus [3]. Dysbiosis impacts gut–brain interconnections through microbial metabolites, including tryptophan derivatives [4]. Therefore, dysbiosis impacts neurological and psychological health [5]. Long-term inflammation, together with the gut barrier, is responsible for carcinogenesis due to certain pathogenic microorganisms, such as *Fusobacterium nucleatum*, *Clostridioides difficile*, and colibactin-producing strains of *Escherichia coli* within the *Enterobacteriaceae* family. For instance, *F. nucleatum* facilitates immune escape and promotes CRC development by creating a pre-metastatic niche in the liver [6].

Similarly, dysbiosis in IBD is characterised by reduced anti-inflammatory bacteria, such as *Faecalibacterium prausnitzii*, and increased pro-inflammatory species that promote inflammation and break gut homeostasis [7]. Celiac disease (CD), or gluten-sensitive enteropathy, explains the well-recognized dynamics of gut microbiota in health maintenance and disease aetiology [8]. CD reduces diversity, which is associated with an overgrowth of pro-inflammatory bacteria, such as those from the phylum *Proteobacteria*, and disruption of intestinal barrier integrity [9]. In a non-invasive approach for observing dietary adherence in mucosa-attacking, active CD, Raman spectroscopy (RS) has been shown to detect metabolic changes related to CD, such as enhanced lipopolysaccharide (LPS) production and altered microbial activity [10].

RS represents a non-invasive and cost-effective alternative to traditional approaches. RS provides a molecular “fingerprint” through inelastic light scattering, which can precisely identify metabolites and microorganisms without destroying the samples’ biological integrity. In contrast to the techniques described above, RS facilitates real-time, functional analysis of the dynamics in microbiota with minimal sample preparation [11]. For instance, RS has been used to identify biomarkers of dysbiosis, such as LPSs and secondary bile acids, which are associated with CRC development. RS allows the analysis of microbial diversity by providing species-specific metabolic signatures and pathogenic activities. RS, therefore, opens perspectives for advancing microbiota research and clinical diagnostics [12]. In this view, restoration of microbial stability by dietary intervention, prebiotics, probiotics, postbiotics, or faecal microbiota transplantation (FMT) underlines the need for sophisticated diagnostic tools like RS.

The current review is focused on RS application in the study of GI health, its potential for discovering novel biomarkers, and its contribution to personalised treatment approaches, considering limitations and future perspectives in clinical and research settings.

## 2. Principles of Raman Spectroscopy

**Figure 1:**
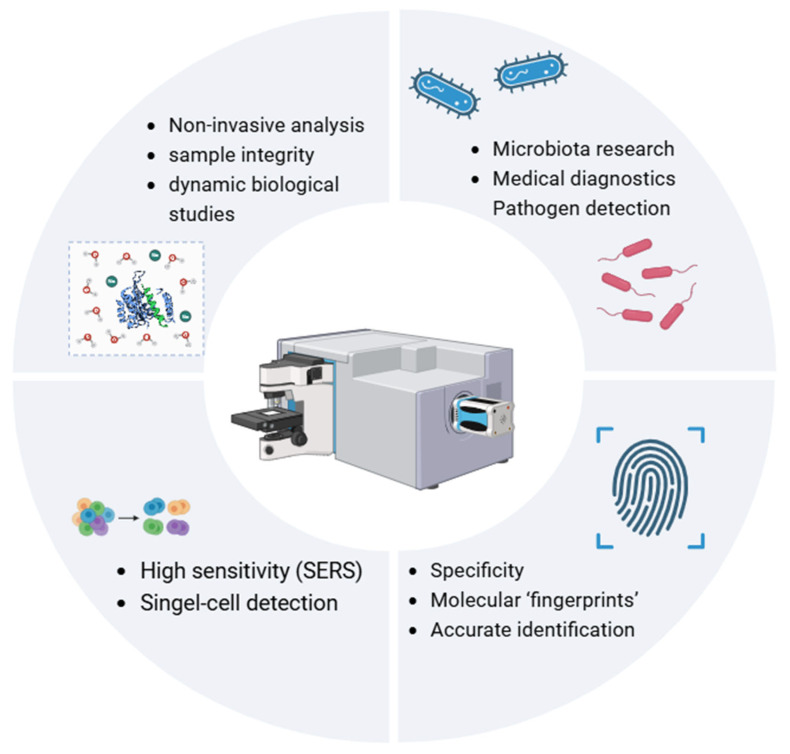
Overview of Raman spectroscopy (RS) principles. SERS—surface-enhanced Raman spectroscopy.

RS is a flexible analytical method relying on inelastic light scattering. In RS, several photons interact with molecular vibrations, creating distinctive spectral “fingerprints” indicating a sample’s molecular makeup [13]. Compared to other techniques, RS can simultaneously analyse various sample types—namely liquids, solids, or gases—requiring minimal preparation [14]. RS is crucial in both biological and medical exploration. The reasons for that are its specificity, sensitivity, and non-invasive characteristics. Every chemical compound creates a unique Raman spectrum that accurately identifies molecules like metabolites and microorganisms. This, in effect, is vital for investigating intercellular interactions and microbial dynamics [15]. Improvements such as surface-enhanced RS (SERS) significantly boost its sensitivity, enabling individual-cell-level detection [16]. This makes RS suitable for high-precision applications, like examining metabolites and recognising pathogens [17]. By preserving the original state of samples, RS supports long-term and live research, making it crucial for understanding dynamic biological systems, including interactions within the microbiota. The principle of operation of RS is shown in Figure 1. Its gentle approach, combined with high precision and sensitivity, establishes it as a key tool in microbiota research, medical testing, and developing new health solutions [18].

## 3. Preparation Methods for Raman Analysis of Biological Samples

Preparing biological samples for RS involves adaptations that depend on the sample’s characteristics and the study’s objectives. The aim is to improve the quality and dependability of Raman spectra while keeping the biological material’s integrity [19]. Analysing biological samples directly, like bacterial cells, is usually possible and causes little disruption, lowering the risk of errors and keeping the sample in a natural condition. This method is helpful for real-time or in situ research. When direct analysis is insufficient, sample enrichment methods separate and purify microorganisms or cell parts from complex biological mixtures [20]. Techniques such as selective filtration and centrifugation help lessen interference from unwanted substances, like proteins or fats, improving the clarity and focus of Raman spectra. These methods are essential for analysing uncommon microorganisms or metabolites. SERS involves nanoparticles, such as gold or silver, to increase sensitivity, which improves Raman signals through localised plasmonic effects [21]. This allows for identifying minute molecules and aids in examining low-concentration biomarkers and molecular interactions at the individual cell level.

**Figure 2:**
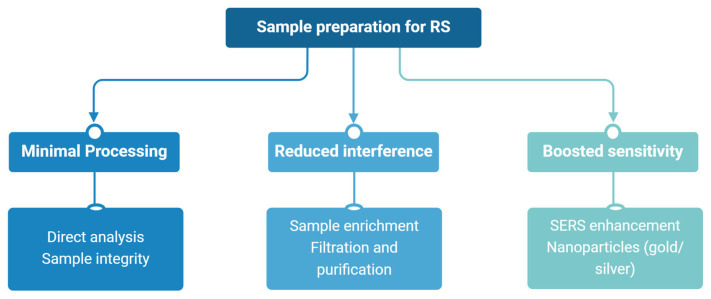
Sample preparation methods for Raman spectroscopy (RS). SERS—surface-enhanced Raman spectroscopy.

Depending on the study’s aims, the selection of preparation methods should balance the desire for minimal processing with the need for sensitivity and specificity [22]. Figure 2 presents the sample preparation methods for RS. Ongoing improvements in these techniques continue to broaden RS’s use, strengthening its role in complex biological studies and its usefulness in microbiota research and medical diagnostics [23].

## 4. Comparison of Raman Spectroscopy with Other Analytical Techniques in Microbiota Studies

RS presents unique advantages compared to other prevalent techniques used in microbiota studies, making it a versatile and supplementary resource in this field. These comparisons are summarised in Table 1, highlighting the unique advantages of RS over other prevalent analytical techniques in microbiota research. Unlike traditional molecular methods, it permits the analysis of samples in their unaltered state, offering direct insights into microbial composition and their chemical contexts. By removing complex preparation steps, the approach retains biological samples’ structural and functional characteristics, distinguishing it from methods such as polymerase chain reaction (PCR), metagenomic sequencing (MS), nuclear magnetic resonance (NMR), and fluorescence microscopy (FM) [24].

Flow cytometry (FC) is also a comprehensive diagnostic method gaining increased awareness in microbiota research [25]. It allows for the quick measurement and classification of microbial groups based on their physical and fluorescence traits. Although FC is excellent for separating microbial subgroups, it does not provide the detailed molecular and chemical specificity that RS offers. However, merging FC with RS can create a complementary method, where separated microbial groups can be further studied at the molecular level with RS, improving the thoroughness of microbiota analysis [26]. Molecular methods like PCR and MS/NGS are established techniques in microbiota research. These approaches effectively identify microorganisms through their genetic material, providing high sensitivity and accuracy, however delivering scarce or no information about microbial viability [27].

Nevertheless, the processes of storage and sampling involved in PCR and NGS can often harm the biological specimen, rendering it unfit for further examination. Moreover, these molecular techniques necessitate complex and time-consuming bioinformatics processes that require specific expertise and computational resources for data analysis. In contrast, RS addresses these challenges by facilitating the direct and non-destructive analysis of samples. This technique identifies microbial species and supplies vital insights into the sample’s chemical composition, enhancing our understanding of the metabolic functions and interactions in the microbiota [28]. NMR spectroscopy is a robust analytical instrument frequently employed in microbiota research, particularly for metabolite profiling [29]. It offers detailed insights into the structure and behaviour of molecules, aiding in identifying and quantifying metabolites in complex biological matrices. However, its application is restricted by the requirement for large sample volumes and prolonged analysis durations.

Additionally, NMR is less sensitive than other methods and high concentrations of analytes are needed for accurate detection. Conversely, RS addresses these limitations by quickly analysing small sample volumes, making it especially effective in examining microorganisms in their natural settings without extensive sample preparation [30]. FM is commonly used in microbiota research, especially for visualising microbial communities, colony forming units (CFU), and monitoring particular molecular targets. This technique employs fluorescent markers to label and identify cellular components, delivering high-resolution spatial data. While FM is essential for its imaging features, external markers can disrupt the studied molecules’ structure and function [31]. Moreover, the process needed to apply fluorescent labels may alter the sample’s natural condition.

In contrast, RS operates without external markers, preserving the inherent characteristics of the biological samples. This marker-free approach reduces the likelihood of inaccuracies, providing a more precise depiction of microbial interactions and chemical compositions [32]. By integrating the benefits of specificity, sensitivity, and being a non-invasive technique, RS enhances these traditional methods while mitigating several limitations. Its ability to perform real-time, non-destructive assessments of microbiota in their natural environments positions it as a vital instrument for investigating the dynamic interactions among microbial communities and their surroundings. As advancements in RS, including SERS and the integration with machine learning, continue to progress, this technique is set to become essential in microbiota research and diagnostics [33].

## 5. Applications of Raman Spectroscopy in Gut Microbiota Studies

RS has emerged as a transformative tool in studying gut microbiota. It provides an influential and non-invasive approach to unravel the complex interplay between microbiota and host health [29]. The diverse applications of RS in microbiota studies are summarised in Table 2, highlighting its role in advancing our understanding of microbial composition and metabolism and their impacts on host health. RS, among other techniques, also enables a more granular understanding of microbial diversity within complex communities [34].

Using machine learning techniques improves RS’s capability to detect microbes. Advanced computational tools, such as neural networks (NNs) and principal component analysis (PCA), assist in rapidly classifying bacterial profiles and recognising specific pathogens or beneficial organisms in microbiota samples. This combination of RS and data analysis methods has proven helpful in diagnosing bacterial infections and studying gut microbiota composition, creating possibilities for immediate, large-scale application in research and healthcare settings [35]. For instance, RS has been applied to identify and classify harmful bacteria within gut microbiota samples, including *Clostridioides difficile* and *Helicobacter pylori*. These bacteria are associated with imbalances in gut flora and digestive problems like IBD, stomach or duodenal ulcers, and gastric cancer [36].

Although metagenomics and 16S rRNA sequencing have been widely adopted for alpha and beta diversity analyses of gut microbiota, traditional approaches are lacking in the identification of live and dead cells, assessing functional activity, and determining real-time dynamics. RS allows for the study of the chemical composition of microbial communities at a functional level, hence addressing some of these challenges. It will enable the detailed profiling of alpha diversity since it aims at distinguishing unique spectral fingerprints attributed to metabolites produced by distinct microbial species [37]. For example, *Faecalibacterium prausnitzii*, an anti-inflammatory commensal, can be traced through specific Raman shifts related to butyrate production.

Similarly, RS can allow beta diversity analyses, comparing metabolite profiles in different samples, and has featured in community-level shifts due to dietary interventions or disease states. RS further enhances species-level identification by overcoming some challenges of spectral overlap with advanced computational approaches, such as machine learning (ML). ML algorithms, including PCA and SVMs, can deconvolute complex spectral data to differentiate even the most closely related microbial species. For instance, RS has been used to probe metabolic signatures that allow for the discrimination of pathogenic strains of *Escherichia coli* from commensal strains, which, generally, is not easy to achieve using conventional sequencing methods [38]. This technique combines RS with machine learning and real-time analysis, making it a strong framework for overcoming the traditional bottlenecks in microbiota research, mainly functional diversity assessment and species-level resolution. These bacteria are associated with imbalances in gut flora and digestive problems like IBD, stomach or duodenal ulcers, and gastric cancer [39].

Additionally, the method has been employed to discover beneficial bacterial types, such as those involved in generating SCFAs, which are crucial for maintaining gut equilibrium. By providing detailed insights into the microbial composition, RS supports a better understanding of microbial alterations and their effects on digestive health [34]. RS is also essential in analysing the substances produced by gut microbiota, offering valuable insights into how microbial communities function and impact host health. The main metabolites are short-chain fatty acids like acetate, propionate, and butyrate that as essential for digestive and immune health [40]. Described acids occur when gut bacteria ferment dietary fibre and assist in energy regulation, support the gut barrier, and decrease inflammation [41]. The Raman spectra from biological samples, such as faeces, urine, or blood, allow for the precise identification and quantification of these metabolites with high accuracy. For example, changes in SCFA profiles detected by RS can indicate dysbiosis or the effectiveness of dietary modifications or probiotic interventions to restore microbial harmony [42].

Furthermore, the method has been employed to monitor the production of other compounds from microbiota, including amino acids, biogenic amines, and phenolic substances, which are linked to host metabolic health and disease [43]. Recent studies have investigated how effective RS is for examining the impact of high-fibre diets on SCFA production and evaluating the success of prebiotic and probiotic supplements. For example, RS has been used to observe the real-time metabolic behaviour of probiotic strains, providing essential insights into their role in alleviating issues like IBS and IBD [44]. This method has also been applied to identify harmful substances related to microbial imbalance, such as LPS and toxic amines, which are connected to the development of gastrointestinal cancers. RS is a vital tool for exploring the interactions between the host and its microbiota by enabling continuous and immediate assessments of microbiota metabolism. This capability presents significant potential for developing personalised treatment strategies, especially concerning dietary interventions and microbiota modulation therapies [45].

## 6. Examples of Raman Spectroscopy Applications in Experimental and Clinical Studies

RS has proven helpful in experimental and clinical research, enabling scientists to examine the changing connections between microbiota and host health. Its application covers various areas, from research on animal models to clinical evaluations and treatment oversight, offering essential insights into how microbiota influences multiple biological processes. In experimental studies, RS has been used to investigate complex microbial groups, such as gut microbiota, in animal models. For instance, this approach has been crucial in studying how dietary changes affect microbiota composition and function. Research with mouse models has demonstrated how high-fat diets alter microbial profiles, resulting in obesity. These results have pointed to a decline in beneficial bacteria, like *Akkermansia muciniphila*, and an increase in harmful species, underscoring the significant role of gut microbiota in regulating metabolism [46]. Moreover, RS has been employed to monitor microbiota responses to treatments in animal studies, enabling researchers to evaluate drug-related changes in microbial diversity and metabolism [34].

Clinically, RS has shown promise in monitoring the response of patients with IBD to anti-TNF therapies using agents such as infliximab and adalimumab. Both agents are widely used treatments for IBD and act to suppress inflammation by neutralising TNF-α, one of the pivotal cytokines of the inflammatory pathways involved in IBD [47]. RS has the potential to parallel changes in other biomarkers of treatment efficacy that include the increased production of SCFAs (that is, butyrate and propionate), which is reflective of improved gut barrier integrity and decreased inflammation. Moreover, RS can identify the decrease in inflammatory-associated metabolites, such as LPS, and microbiota diversity changes, such as the recovery of anti-inflammatory properties species, such as *Faecalibacterium prausnitzii* [48]. Beyond anti-TNF therapies, RS can also be used to investigate other biologics, including vedolizumab, an anti-integrin agent that modulates immune cell trafficking to the gut, and ustekinumab, which targets interleukin-12/23 pathways [49]. RS could provide real-time feedback on how such medications alter the gut microbiota and metabolite profiles to optimise treatment strategies and personalise therapy.

Such understanding is also vital for developing targeted therapies that alter microbiota. In clinical research, RS has effectively identified signs of microbial alterations associated with various digestive disorders [38]. For patients with IBDs, such as Crohn’s disease and ulcerative colitis, this method has been used to detect shifts in microbiota composition and their associated metabolic byproducts, including SCFAs and bile acids. These signs indicate disease status and provide insights into the mechanisms behind inflammation and gut barrier integrity [27]. By monitoring microbial composition and metabolism changes, RS has been utilised to track patients’ responses to biological therapies, such as anti-TNF medications. This capability supports personalised medicine strategies by adjusting treatment approaches based on individual microbiota profiles. In probiotic research, RS has been used to evaluate probiotic strains’ colonisation and metabolic activities in patients’ digestive systems [43]. For example, studies have tracked the presence of probiotic *Bifidobacterium* and *Lactoplantibacillus* species in stool samples, assessing their role in restoring microbiota balance.

The method has also enabled the examination of SCFA production associated with probiotic use, presenting a functional output for microbial activity and its impact on gut health. This non-invasive method is especially valuable for long-term studies, where repeated assessments are essential to observe the effects of probiotics or prebiotics on microbiota. The use of RS in these experimental and clinical environments emphasises its versatility and potential to advance microbiota research. By providing detailed descriptions of microbial communities and their interactions with the host, the method acts as a powerful tool for revealing the role of microbiota in health and disease, paving the way for innovative diagnostic and treatment strategies [29].

## 7. The Potential of RS in Identifying Biomarkers of GI Health and Disease

RS creates opportunities for detecting disease indicators implicated in GI health. By enabling detailed chemical analysis of biological samples, this technique offers insights into the metabolic and compositional changes in gut microbiota that indicate various disease states. With its ability to detect metabolites from microbiota, such as SCFAs, fats, and proteins, RS is a valuable tool for assessing gut health and identifying early signs of disease progression [50]. Changes in SCFA levels, such as reduced butyrate, have been associated with conditions like IBD and CRC [48]. RS allows for precise detection and quantification of SCFAs in biological samples like stool and blood, providing a non-invasive means to monitor gut microbial health and activity. Besides SCFAs, RS can detect fats, proteins, and other compounds essential for identifying disease-specific markers [35]. For example, this technique has been employed in CRC research to discover chemical indicators related to tumour growth, such as increased levels of harmful substances, including secondary bile acids, phenolic compounds, and biogenic amines. Apart from CRC, RS has also been used to diagnose and follow up other GI malignancies, such as gastric and oesophageal adenocarcinomas. In gastric adenocarcinoma, RS can detect metabolic changes such as increased lactate—as a sign of enhanced glycolysis (Warburg effect)—and a changed protein-to-lipid ratio. Some spectral biomarkers, including increased peaks associated with nucleic acids and collagen, allow discrimination between normal, dysplastic, and malignant tissues [51]. RS has been utilized in the study of oesophageal adenocarcinoma to analyze indicators linked to oxidative stress and lipid peroxidation, which both signify cellular damage and cancer progression.

Furthermore, RS can identify secondary bile acids such as deoxycholic acid and lithocholic acid, which contribute to establishing a cancer-promoting environment in gastric and colorectal cancers. Merging RS with advanced machine learning techniques enhances the evaluation of these spectral biomarkers, resulting in better accuracy in diagnosis, monitoring of tumor growth, and evaluation of treatment effects. For instance, RS has been intraoperatively applied during surgical resections to provide real-time feedback about tumour margins, thus enhancing the precision of oncological interventions [52]. These materials arise due to an imbalanced allocation of microbiota and can encourage cancer through mechanisms like DNA damage, persistent inflammation, and disruption of cell signaling pathways.

In addition to its diagnostic function, RS also supports the evaluation of different therapeutic approaches, including probiotics, prebiotics, or postbiotics, along with dietary modifications, by analyzing their impact on the makeup and metabolic functions of microbiota. This method provides a rapid, non-invasive way to measure the effectiveness of these therapies by tracking changes in beneficial metabolites or potentially harmful microbial products [53]. Studies have indicated that RS can be utilised to monitor SCFA recovery and reinstate microbiota diversity following probiotic use. This ability could be especially beneficial in future personalised medicine protocols, where treatments are tailored to each patient’s unique microbiota profile. Another promising application of RS is its capacity to detect early signs of GI diseases, facilitating preventive strategies. For instance, in IBD patients, RS has been used to discover metabolic alterations before the onset of clinical manifestations. This early detection capability can improve disease management by enabling timely treatment interventions [48].

In summary, RS is a powerful and flexible method for detecting disease markers associated with GI health. This technique deepens our knowledge of the fundamental processes underlying gut disorders by delivering precise chemical insights into the metabolism and structure of microbiota. It plays an essential role in crafting focused diagnostic and treatment approaches. With its swift, non-invasive capabilities and proficiency in analysing diverse biological samples, RS is vital for progressing research and clinical practices related to GI health.

## 8. Clinical Applications of Raman Spectroscopy

RS has emerged as a valuable approach for identifying and tracking health-related conditions in clinical settings, offering rapid and non-invasive evaluations of gastrointestinal problems. Its capability to create intricate molecular “signatures” of biological materials such as stool, bodily fluids, and tissue samples has been essential in identifying, observing, and handling different gastrointestinal disorders. New technological improvements, such as the creation of handheld Raman spectrometers suitable for testing at the point of care, have further improved the adaptability of this method. One of RS’s most promising clinical applications is identifying GI disorders [54]. By analysing the chemical composition of biological samples, Raman spectroscopy can differentiate pathological conditions that share similar symptoms but have distinct biochemical origins. For instance, this technique has effectively differentiated between IBD and IBS.

This distinction is achieved by identifying variations in metabolite profiles, including SCFAs and lipids, that are significantly altered in these conditions. In CRC diagnosis, RS has been used to identify specific cancer markers, encompassing altered protein and fat ratios in tissues [55]. In GI tumours, particularly adenocarcinomas, RS has demonstrated the ability to detect specific molecular alterations associated with tumour development and progression. These include elevated levels of secondary bile acids such as deoxycholic acid, which are known to promote DNA damage and tumorigenesis, as well as changes in lipid and protein composition within tumour tissues [56]. Furthermore, RS has been employed to distinguish between healthy, dysplastic, and malignant tissues based on distinct Raman spectral patterns. For example, adenocarcinomas exhibit increased intensity of peaks associated with nucleic acids, lipids, and proteins, reflecting heightened cellular proliferation and metabolic reprogramming [57]. RS has also been used to detect metabolic byproducts like phenolic compounds and biogenic amines, which are elevated in adenocarcinomas due to dysbiosis and inflammation [58]. These capabilities position RS as a powerful tool for the early detection, diagnosis, and monitoring of adenocarcinomas, with the potential for integration into personalised diagnostic approaches. These alterations indicate tumour development and are challenging to detect using conventional methods. Accurately identifying such markers makes RS a valuable instrument for early cancer detection and monitoring. This differentiation is made possible by detecting changes in metabolite patterns, which include short-chain fatty acids and lipids that are notably modified under these circumstances. In the diagnosis of colorectal cancer, RS has been utilised to pinpoint cancer indicators, including secondary bile acids like deoxycholic acid, phenolic substances, and biogenic amines such as putrescine and cadaverine, all of which are associated with the development of tumours. These indicators play a role in tumour growth through various processes, including ongoing inflammation, genetic damage, and disturbances in cellular signalling systems [59].

Furthermore, altered protein-to-fat ratios within tissues, identified through Raman spectroscopy, act as another significant marker for colorectal cancer. Precisely identifying these indicators positions RS as a crucial tool for the early detection and ongoing monitoring of cancer [60]. Additionally, its ability to analyse samples without causing damage ensures that specimens remain intact for further research and validation efforts. Moreover, its non-destructive nature allows for the preservation of samples, facilitating additional analyses and validation studies [34].

RS also excels at monitoring the effectiveness of medical and dietary interventions in real-time. This technique has been employed in IBD treatment to observe shifts in gut microbiota composition and their associated metabolic byproducts, such as SCFA production, after biological therapies or probiotic administration. These real-time assessments provide essential feedback on treatment efficacy, enabling physicians to modify therapeutic strategies for individual patients [61]. In nutritional research, RS has been applied to explore how high-fibre diets affect gut microbiota. This approach provides understanding into how changes in diet support gut wellness by assessing the growth of helpful bacteria and the related rise in SCFA production. These findings highlight the possibilities of Raman spectroscopy as a non-invasive method for assessing the long-term impacts of eating habits on microbiota and general health [29]. Recent advancements in technology have resulted in smaller RS systems, leading to the creation of portable Raman spectrometers. These instruments allow the diagnostic features of RS to be brought directly into medical settings, facilitating immediate testing that removes the need to send samples to centralized labs.

Portable Raman spectrometers allow for quick, on-site examination of biological samples, making them perfect for use in microbiota diagnostics, monitoring metabolic changes, and spotting disease markers. Integrating portable Raman devices into clinical workflows significantly impacts real-time therapeutic decision making [21]. By delivering immediate results, these instruments assist healthcare providers in tailoring treatments based on individual patient information, enhancing the accuracy and effectiveness of interventions. For example, portable Raman spectrometers have been used to identify dysbiosis-related markers in stool samples, enabling quick adjustments to diet or medication treatments [62]. The clinical applications of RS indicate its ability to change the diagnosis and treatment of gastrointestinal disorders. RS provides remarkable versatility and precision by enabling the differentiation of complex pathological states, monitoring treatment success, and supporting point-of-care testing. As technological advancements continue to improve the accessibility and feasibility of this technique, its application in clinical settings is likely to increase, supporting the development of personalised medicine and improved patient outcomes [63].

## 9. Limitations and Challenges in Clinical Applications of Raman Spectroscopy

Even though it has potential as a diagnostic technique in clinical and microbiota research, RS faces significant challenges that must be addressed for broader application in healthcare. Due to low inelastic photon scattering, the weak Raman signal decreases sensitivity in complex biological samples where overlapping signals obscure crucial information [64]. SERS enhances signals using metallic nanoparticles, but this approach adds more steps in preparation, making it more complex and variable. Biological spectra also create a challenge because of their intricacy, requiring advanced computational tools like machine learning to analyse overlapping signals from proteins, lipids, and nucleic acids [65]. However, the need for substantial infrastructure and expert knowledge limits clinical availability.

The high costs of Raman systems, including portable versions, further restrict their use to well-funded institutions. At the same time, the lack of standardised methods for sample preparation, data gathering, and spectral interpretation results in inconsistent outcomes and hinders clinical reliability [11]. Efforts to overcome these challenges include combining Raman spectroscopy with complementary methods like mass spectrometry, utilising machine learning to simplify data analysis, and advancing standardisation to improve reliability. These advancements could establish RS as a vital tool in personalised medicine, offering rapid, non-invasive diagnostics tailored to individual patients. In conclusion, while Raman spectroscopy faces considerable challenges, ongoing technological advancements, computational strategies, and standardisation efforts create opportunities for its broader acceptance in clinical settings [1]. By addressing these challenges, Raman spectroscopy could become a key component of future diagnostic and treatment approaches.

## 10. Key Conclusions on the Application of Raman Spectroscopy in Gut Microbiota Studies

RS has become a flexible and innovative analytical method, resulting in significant progress in studying the gut microbiota. Its capability to produce distinct molecular spectra makes it an essential tool for identifying microorganisms, examining microbial metabolites, and monitoring changes in microbiota composition due to various interventions [66]. These interventions include dietary changes, medical therapies, and the use of probiotics, prebiotics, or postbiotics, all of which are vital for investigating the dynamic relationships between microbiota and host health. What distinguishes RS is its capacity for conducting non-invasive analyses directly from biological samples without requiring extensive preparation or destruction [34]. This feature is particularly significant for studies focused on gastrointestinal health, where preserving sample integrity is crucial for long-term or real-time research.

Furthermore, its specificity and sensitivity have been critical in distinguishing complex gastrointestinal disorders, such as inflammatory bowel disease and colorectal cancer, by identifying condition-specific biomarkers. Some of these biomarkers for specific health-related diseases have demonstrated changes in the profiles of SCFAs, mainly decreases in butyrate, which seem to be linked with IBD and CRC [67]. Moreover, increases in secondary bile acids such as deoxycholic acid and lithocholic acid are associated with tumour development in CRC [56]. Substances of phenolic origin and biogenic amines, including cadaverine and putrescine, represent markers of dysbiosis and active inflammation, underlining their contribution to gastrointestinal disorders [58]. Changes in microbial diversity are essential markers too, such as a decrease in helpful bacteria like *Faecalibacterium prausnitzii* or *Akkermansia muciniphila*, which can be detected by RS [68]. These attributes underscore Raman spectroscopy’s potential to connect advanced microbiota research with clinical diagnostic uses [29].

RS offers distinct insights into the by products produced by gut microbiota, particularly short-chain fatty acids that are essential for maintaining intestinal health [1]. Fatty acids such as acetate, propionate, and butyrate are vital in promoting epithelial integrity, impacting immune responses, and managing inflammation. The changes in SCFA profiles revealed through RS can serve as early indicators of microbial alterations leading to dysbiosis and its associated health impacts, including DGBIs and IBD. In addition to SCFAs, RS has been utilised to explore other metabolites like amino acids, bile acids, and phenolic compounds, providing a broader overview of microbiota activity. Moreover, RS could facilitate the identification of specific biomarkers related to microbial alterations, such as variations in microbial diversity and the synthesis of metabolic products [69]. RS gives unique insight into the metabolic products, such as SCFAs, of gut microbiota, which are essential for maintaining a balanced gastrointestinal system. RS may allow identifying specific biomarkers. For example, RS can reveal variations in metabolic byproducts such as indole and skatole. These compounds arise from the microbial fermentation of proteins and are associated with dysbiosis in disorders like IBD [70]. It also can detect heightened concentrations of LPS, a biomarker of heightened gut permeability and systemic inflammation well documented in inflammatory bowel diseases [71]. Moreover, RS might reveal changes in microbial-derived metabolites, such as tryptophan and its downstream products, that play important roles in communication in the gut–brain axis and have recently been implicated in disorders ranging from functional GI to mood disorders [72]. The capability to identify diverse metabolites underlines the versatility of RS as a diagnostic tool in microbiota-related research. This capability is significant for understanding the mechanisms underlying microbiota-related disorders. For example, detecting elevated levels of harmful metabolites like secondary bile acids and phenols in CRC highlights the significance of RS in studying microbiota-driven disease development and progression. By monitoring microbiota responses to various interventions, RS supports the creation of personalised treatment strategies tailored to an individual’s specific microbiota profile.

Combining RS with omics methods, including metagenomics, metabolomics, transcriptomics, and proteomics, holds significant potential for enhancing personalised medicine [73]. Researchers could better understand host–microbiota interactions by integrating Raman’s capability to analyse molecules using vast information from omics techniques. This integrated method may allow for the discovery of new biomarkers, provide better understanding of disease processes, and assist in creating more precise and tailored treatment strategies. The advancement of portable Raman spectrometers has expanded the use of this technology in medical environments. These compact devices allow for rapid, non-invasive microbiota analysis at the care location, simplifying access to tests and facilitating immediate treatment choices. Healthcare providers can utilise portable Raman spectrometers to assess microbiota-related biomarkers directly from patient samples, such as stool or blood, providing quick feedback on gut health [13]. This capability is particularly crucial for managing chronic conditions like IBD, where monitoring treatment responses is vital for enhancing treatment outcomes. Moreover, advancements in AI and ML in spectral analysis are expected to revolutionise the function of RS in various applications. AI-driven models can interpret complex spectral data to identify patterns and connections that would be difficult to discern manually. This development will streamline microbiota profiling and biomarker identification, reducing the reliance on specialised expertise and enhancing the application of RS in clinical settings [74]. RS could lead to the development of innovative, personalised treatments aimed at modulating GI microbiota. For example, Raman-based assessments may assist in creating prebiotic or probiotic products tailored to an individual’s specific microbial profile and metabolic needs. This approach could open new avenues for treating GI diseases and general health concerns influenced by gut microbiota, such as metabolic and neurodegenerative disorders [75].

## 11. Conclusions

To conclude, RS is an innovative method for studying gut microbiota that provides a fresh understanding of how the microbiome influences digestive well-being and GI disease. Its distinct blend of precision, non-invasive nature, and real-time analysis capabilities make it an essential resource for medical and research purposes. The continuous improvement of RS, especially when integrated with other omics and AI technologies, could transform diagnostic methods and treatment protocols, elevating personalised medicine to the next level. RS is anticipated to aid significantly in the progress of healthcare by connecting clinical practice with microbiota analyses.

## Figures and Tables

**Table 1:** Comparison of Raman spectroscopy (RS) with other analytical techniques.

Technique	Advantages	Limitations	Cost
Raman Spectroscopy (RS)	Non-invasive, minimal sample preparation, detects chemical composition.	Weak signal, complex spectra require advanced analysis.	Moderate with portable options.
PCR/Metagenomics	High sensitivity and specificity for identifying genetic material.	Expensive and requires extensive bioinformatics tools.	High due to sequencing and bioinformatics costs.
NMR Spectroscopy	Provides detailed structural and dynamic molecular data.	High sample quantity and long analysis times needed.	Very high due to equipment and operation costs.
Fluorescence Microscopy (FM)	Visualises specific molecules using fluorescent markers.	Requires markers that may interfere with native structures.	Moderate but requires marker-specific reagents.
Flow Cytometry (FC)	Rapid quantification and sorting of microbial populations based on physical/fluorescence properties.	Lacks detailed molecular and chemical specificity; requires fluorescence markers.	High due to equipment and consumables.

PCR—Polymerase Chain Reaction; NMR—Nuclear Magnetic Resonance.

**Table 2:** Applications of Raman spectroscopy in microbiota studies.

Application	Description
Identification of Microorganisms	Differentiating bacterial species and strains based on unique Raman spectral fingerprints.
Analysis of Microbial Metabolites	Detection of key metabolites such as SCFAs, amino acids, and phenolic compounds.
Monitoring Changes in Microbiota	Tracking microbiota responses to dietary, pharmacological, or probiotic interventions.
Biomarker Discovery	Identifying markers of microbial alterations/dysbiosis/in conditions like DGBI, IBD, and colorectal cancer.
Real-time Metabolic Studies	Studying metabolic activities dynamically in microbiota under various conditions.

DGBI—Disorders of Gut–Brain Interaction, IBD—Inflammatory Bowel Disease; SCFAs, Short Chain Fatty Acids.

## Data Availability

Data sharing does not apply to this article as no new data were created or analysed in this study.
